# Linguistic scope-based and biological event-based speculation and negation annotations in the BioScope and Genia Event corpora

**DOI:** 10.1186/2041-1480-2-S5-S8

**Published:** 2011-10-06

**Authors:** Veronika Vincze, György Szarvas, György Móra, Tomoko Ohta, Richárd Farkas

**Affiliations:** 1 Research Group on Artificial Intelligence, Hungarian Academy of Sciences, Szeged, Hungary; 2 UKP Lab, Technische Universität Darmstadt, Darmstadt, Germany; 3 Department of Informatics, University of Szeged, Szeged, Hungary; 4 Tsujii Laboratory, University of Tokyo, Tokyo, Japan; 5 lnstitut für Maschinelle Sprachverarbeitung, Universität Stuttgart, Stuttgart, Germany

## Abstract

**Background:**

The treatment of negation and hedging in natural language processing has received much interest recently, especially in the biomedical domain. However, open access corpora annotated for negation and/or speculation are hardly available for training and testing applications, and even if they are, they sometimes follow different design principles. In this paper, the annotation principles of the two largest corpora containing annotation for negation and speculation – BioScope and Genia Event – are compared. BioScope marks linguistic cues and their scopes for negation and hedging while in Genia biological events are marked for uncertainty and/or negation.

**Results:**

Differences among the annotations of the two corpora are thematically categorized and the frequency of each category is estimated. We found that the largest amount of differences is due to the issue that scopes – which cover text spans – deal with the key events and each argument (including events within events) of these events is under the scope as well. In contrast, Genia deals with the modality of events within events independently.

**Conclusions:**

The analysis of multiple layers of annotation (linguistic scopes and biological events) showed that the detection of negation/hedge keywords and their scopes can contribute to determining the modality of key events (denoted by the main predicate). On the other hand, for the detection of the negation and speculation status of events within events, additional syntax-based rules investigating the dependency path between the modality cue and the event cue have to be employed.

## Background

In natural language processing (NLP) – and in particular, in information extraction (IE) – many applications seek to extract factual information from text. In order to distinguish assertions from unreliable/uncertain information and negated statements, linguistic devices of negation or hedges have to be identified. Applications should handle detected modified parts in a different manner. A typical example is protein-protein interaction extraction from biological texts, where the aim is to mine text evidence for biological entities that are in a particular relation with each other. Here, while an uncertain relation might be of some interest for an end-user as well, such information must not be confused with factual textual evidence (reliable information).

There are several available negation and hedge detection systems (usually for the clinical and biological domains). The first systems were fully hand-crafted [1-3] without any empirical evaluation on a dedicated corpus. Recently, there have been several corpora published with manual annotation and several rule-based systems have been developed and evaluated on them [4,5].

Recent approaches exploit machine learning models. Medlock & Briscoe [6] used single words as input features in order to *classify sentences* from biological articles (FlyBase) as speculative or non-speculative based on semi-automatically collected training examples. Szarvas [7] extended their methodology to use n-gram features and a semi-supervised selection of the keyword features. Using BioScope [8] for training and evaluation, Morante et al. [9] developed *in-sentence scope detectors* for negation and speculation following a supervised sequence labeling approach, while Özgür and Radev [10] constructed a rule-based system that exploits syntactic patterns. BioScope is also the source of training and evaluation datasets of the CoNLL-2010 Shared Task [11]. Several related works have also been published within the framework of The BioNLP’09 Shared Task on Event Extraction [12], where a separate subtask was dedicated to predicting whether the recognized *biological events are under negation or speculation *[*4*].

In this paper we focus on corpora annotated for negation and speculation. There are several available corpora outside the biomedical domain (e.g. FactBank [13], Wikipedia weasels [11]) as well. However, we deal here with biological information extraction and to our best knowledge, the following related corpora have been constructed for this domain:

• The Genia Event corpus [14] which annotates biological events with negation and two types of uncertainty (9372 sentences).

• The Biolnfer corpus [15] where biological relations are annotated for negation (1100 sentences in size).

• The BioScope corpus [8], which includes three types of texts from the biomedical domain – namely, radiological reports, biological full papers and abstracts from the Genia corpus – annotated for both negation and hedge keywords and their linguistic scopes (20924 sentences).

• The system developed by Medlock & Briscoe [6] made use of a corpus consisting of six papers from genomics literature in which 1537 sentences were annotated for speculation. These texts – with re-annotation – are also included in BioScope.

• Shatkay et al. [16] describe a database where 10000 biomedical sentences are annotated for polarity and three levels of certainty.

In the corpora Genia Event and BioInfer, biological concepts (relations and events) have been annotated for negation and – in the case of Genia Event – for hedging as well, but linguistic cues (i.e. which keyword modifies the semantics of the statement) have not been annotated for them. In the last two corpora, speculative annotation can be found on the sentence level.

In contrast to those, BioScope was not fine-tuned for information extraction tasks but it contains linguistic annotation for hedge and negative cues and their in-sentence scope as well. Its chief objective is to investigate these language phenomena in a general, task-independent and linguistically-oriented way. Automatically recognized in-sentence scopes (i.e. the negated or hedged text spans) are important for many natural language processing applications. For instance:

• in clinical document classification tasks [17,18], the goal is to assign labels to medical documents according to factual statements about the patient in question. Here the removal (or separate handling) of hedged or negated text spans has a great contribution in the training and prediction phases as well.

• In information retrieval the query mentions under hedging can be ranked lower,

• in machine translation the extension of negation or speculation scopes has to be precisely known in order to translate meaning adequately.

Although the BioScope corpus consists of clinical and biological documents, its annotation guidelines do not contain any domain-specific instruction. Councill et al. [19] employed BioScope as training corpus for detecting negated scopes for opinion mining from product reviews, which illlustrates the applicability of BioScope’s annotations in a different task and domain.

In the following sections, the hedge and negation annotation principles of BioScope and Genia Event are compared, resolution strategies for the differences are offered and we discuss how BioScope can contribute to identifying “new knowledge” in biomedical papers.

## Methods

In this paper we quantitatively compare the negation and speculation annotations of the BioScope and Genia Event corpora. We investigated sentences that occur in both corpora, i.e. the intersection of the two corpora containing 958 abstracts and 8942 sentences (abstracts that were not segmented in the same way on the sentence level in the two corpora were neglected) was used. This corpus contains 1287 negation and 1980 speculation BioScope scopes (376 nested scopes) while 2123 non-exist and 1475 probable Genia events (200 events have both labels).

As for negation, events with at least one clue occurring within a negative scope in BioScope and being annotated as non-exist in Genia Event were considered as cases of agreement. With regard to speculation, events with at least one clue within a speculative scope in BioScope and being marked as probable in Genia Event were accepted as cases of agreement. Mismatches included events with different labels in the two corpora (e.g. an event labeled as negative in Genia Event and speculative in BioScope) on the one hand, and events annotated only in one of the corpora on the other hand.

In order to understand the differences between the annotation principles and to investigate the possible contribution of the BioScope annotation to Genia event modality detectors, we randomly sampled 200 sentences from the intersection of the two corpora. This sampling consists of 50 sentences where events are marked to be negated by Genia and none of its arguments was included in a BioScope negation scope and 50 sentences where at least one of the arguments of an event was under a BioScope negation scope and marked as existing by Genia (50+50 sentences were selected for speculation analogously). By manual inspection of this sample we thematically categorized these differences.

## Results

### Annotation principles

#### BioScope annotation

When annotating keywords and their scopes in the BioScope corpus [8], the corpus builders followed a min-max strategy. When marking the keywords, a minimalist strategy was followed: the minimal unit that expresses hedging or negation is marked as a keyword. Special attention is paid to the case of complex keywords, that is, words that express uncertainty or negation together, but not on their own (either the semantic interpretation or the hedging strength of its subcomponents are significantly different from those of the whole phrase).

The scopes of negative and speculative keywords are extended to the largest syntactic unit possible. Thus, annotated scopes always have the maximal length. (Inter-annotator agreement rates are available at the corpus website: http://www.inf.u-szeged.hu/rgai/bioscope.) In the next example, *however* is not affected by the hedge cue but it should be included within the scope, otherwise the keyword and its target phrase would be separated (scopes are marked by brackets and keywords are bold):

[Atelectasis in the right mid zone is, however, ***possible***]*.*

That is why the corpus builders preferred to include every possible element within the scope rather than exclude elements that should probably be included. As for annotating, the most important thing to consider is that hedging or negation is determined not just by the presence of an apparent cue: it is rather an issue of the keyword, the context and the syntactic structure of the sentence taken together. The scope of a keyword can be determined on the basis of constituency grammar. The scope of verbs, auxiliaries, adjectives and adverbs usually extends to the right of the keyword. In the case of verbal elements, i.e. verbs and auxiliaries, it ends at the end of the clause (if the verbal element is within a relative clause or a coordinated clause) or the sentence, hence all complements and adjuncts are included, in accordance with the principle of maximal scope size. In the case of elliptic sentences, the scope of the negative keyword may be deleted as in:

This decrease was seen in patients who responded to the therapy as well as in those who did [***not***].

In these cases, the scope contains only the keyword.

#### Genia Event modality annotation

The Genia Event corpus was primarily designed for (biological) event annotation [14] and the database contains annotation for uncertainty and negation on the level of events. The annotation scheme focuses on events, and arguments of events can occasionally be found across clause boundaries, typically due to anaphora or coreference (out of 35419 Genia events used in our experiment, 1127 referred to an external event and 2076 clues are arguments of an event expressed in another sentence (mostly cluetypes *theme* (1447 instances, 70%) and *cause* (619 instances, 29.8%)).

As for uncertainty, events can have three labels in the corpus: certain, probable and doubtful. Events are marked as doubtful if they are under investigation or they form part of a hypothesis, etc. An example (event arguments are underlined in our examples) for a doubtful event is provided here:

We then investigated if HCMV binding also resulted in the translation and secretion of cytokines.

Events are considered probable if their existence cannot be stated for certain. An example of a probable event is shown here:

Together, this evidence strongly implicates BSAP in the regulation of the CD19 gene.

The attribute certain is chosen by default if none of the two others hold: an event the existence of which cannot be questioned in any way.

As for negation, events are marked with the labels exist or non-exist. An example for a negated event is shown below:

Analysis of Tax mutants showed that two mutants, IEXC29S and IEXL320G, were unable to significantly transactivate the c-sis/PDGF-B promoter.

In the corpus, no explicit marking of either the keywords or the scope of negation and hedging can be found.

### Number of disagreements

Table 1 shows the number of cases of agreement and disagreement between the two corpora (agreement rate: 48%). The numbers in column TP (true positive) denote instances which are considered in the same way in both corpora. The numbers in column BPGN refer to cases where in BioScope any clue of a Genia event is under a negative / speculative scope, however, in Genia Event, it is not. As opposed to this, in column GPBN, the numbers show cases where Genia contains some speculative / negative annotation for any argument of the event but BioScope does not.

**Table 1:** Numbers of agreement and disagreement between BioScope and Genia Event.

	TP	BPGN	GPBN
negation	1554	1484	569
probable	1295	3761	180

Table 1 shows the number of disagreement between BioScope and Genia Event.

#### Categorization of differences

In this section, mismatches in annotation between the Genia Event and the BioScope corpora are presented. Systematic differences are categorized on the basis of a possible solution aiming to resolve the mismatch, and subtypes of these categories are illustrated with examples along with their estimated frequencies based on a random sample of 200 annotation differences (see Table 2).

**Table 2:** Frequency of mismatch categories

	BPGN	GPBN
event within event	68%	–
syntax	7%	3%
lexical semantics	20%	72%
morphological negation	–	14%
annotation error	5%	11%
TOTAL	100%	100%

Table 2 shows the estimated ratio of mismatch categories between BioScope and Genia Event.

##### Event-centered vs. linguistic annotation

An essential difference in annotation principles between the two corpora is that Genia Event follows the principles of Event-centered annotation [14] while BioScope annotation does not put special emphasis on events as it aims a task-independent modeling of speculation and negation. Event-centered annotation means that annotators are required to identify as many biological events as possible within the sentence then label each separately for negation and speculation. Events are usually expressed by verbs, however, (deverbal) adjectives and nouns can also refer to events. Consider the following example:

Calcineurin acts in synergy with PMA to inactivateI kappa B/MAD3, an inhibitor of NF-kappa B.

This sentence describes two events, the *inactivation of I kappa B/MAD3 by Calcineurin* and the *inhibition of NF-kappa B by I kappa B/MAD3.*

From a linguistic point of view, an event is understood as a predicate together with its arguments and the role of the predicate can be fulfilled by a verb, a noun, or an adjective in the text. In contrast to this, BioScope is not event-oriented in the above sense. Instead, verbs play a central role, i.e. a verb and its arguments form one event in BioScope as well. Accordingly, the above sentence refers to one event in BioScope and *inhibitor* is not considered as a predicate.

As a consequence, there are much more events in Genia than in BioScope. The multiplicity of events in Genia Event and the maximum scope principle exploited in BioScope taken together often yields that a Genia event falls within the scope of a BioScope keyword, however, it should not be seen as a speculated or negated event on its own. Here we provide an illustrative example:

In summary, our data [***suggest*** that changes in the composition of transcription factor AP-1 is a key molecular mechanism for increasing IL-2transcription and may underlie the phenomenon of costimulation by EC].

According to the BioScope analysis of the sentence, the scope of *suggest* extends to the end of the sentence. It entails that although in Genia it is only the events *is a key molecular mechanism* and *underlie the phenomenon* that are marked as probable, the events *changes*, *increasing*, *transcription* and *costimulation* are also included in the BioScope speculative scope. Thus, within this sentence, there are six Genia events out of which two are labeled as probable, however, in BioScope, all six are within a speculative scope, resulting in two cases of agreement and four cases of disagreement. Concerning the whole corpora, the large number of BPGN cases (see Tables 1 and 2) can be explained in a similar way.

##### Syntactic issues

Some of the mismatches in annotation can be traced back to syntax. For instance, the treatment of subjects remains problematic since in BioScope it is only the complements that are usually included within the scope of a keyword (that is, subjects are not with the exception of passive constructions and raising verbs) in contrast to Genia where events are argument-centered (i.e. complements and subject are considered) as in:

Both c-Rel and RelA induced jagged1 gene expression, whereas a mutant defective for transactivation did [***not***].

In this example, no argument of the event denoted by *induced* is under the BioScope scope, which yields a case of disagreement.

With regard to the problem concerning the treatment of subjects, the dependency parse of the sentence/clause might help the correct identification of the modality of the events. We can apply the following rule: if a verb that functions as the trigger word for an event is negated or hedged, all its children in the dependency tree (including the subject as well) are to be included in the scope of the modifier. In this way, instances of mismatch when it is only the subject that is within the scope of the modifier (e.g. in the case of elliptic sentences) can be eliminated from the GPBN set.

##### Semantic issues

There are some cases where the difference in annotations originates from conceptual discrepancies. These differences can hardly be resolved without harmonizing the annotation principles behind the corpora and re-annotating the data, however, the most typical cases are presented here. Events labeled as doubtful in Genia Event are rarely annotated as speculative in BioScope. In Genia Event, the investigation, examination, study, etc. of a phenomenon does not necessarily mean that the phenomenon exists. However, in BioScope this aspect is neglected and phenomena being under investigation, examination, etc. are only marked as instances of speculation if they are within the scope of a speculative keyword (e.g. *whether*)*.* As only 17% of doubtful Genia event clues is under speculation scope, we focus just on the probable class during our comparison.

There are some examples of mismatch where a generalization or a widely accepted claim is stated. Grammatically, these sentences usually occur in the passive voice without explicitly marking the agent (i.e. the one whom the claim originates from). Such sentences are instances of weaseling [20], and are annotated as probable events in Genia, however, in BioScope they are not as they express a different type of uncertainty: it is the exact source of the opinion that is missing rather than the factuality of the event (it is known that some hold this opinion but it is unknown who they are). It is a kind of uncertainty expressed at the discourse level as opposed to uncertainty on the semantic level. An example for a weasel sentence is shown below:

Receptors for leukocyte chemoattractants, including chemokines, are traditionally considered to be responsible for the activation of special leukocyte functions such as chemotaxis, degranulation, and the release of superoxide anions.

Weasel sentences and cue phrases can be automatically detected by employing machine learnt models. For instance, the CoNLL-2010 Shared Task dataset [11] includes a corpus dedicated to weasel detection in Wikipedia articles. We suppose that the phenomenon of weasel is domain-independent hence the model trained on Wikipedia could be adequately applied for (biological) scientific publications as well. 

Sometimes an event is marked as negation in BioScope but not in Genia:

[***Lack of*** full activation of NF-AT] could be correlated to a dramatically reduced capacity to induce calcium flux and could be complemented with a calcium ionophore.

As *lack* is understood as ‘the state of not having something’, it denotes negation, i.e. the non-existence of the following NP complement, that is why it is marked as a negative keyword in BioScope. However, in Genia, ‘lack of something’ is understood as negation of status, not negation of an event. Hence here the class type of the event is negative regulation but the event itself is assertive (out of 4347 negative regulations in Genia 4164 are assertive, some of which are annotated as negative in BioScope due to semantically negative keywords).

Another case of conceptual discrepancy is morphological negation, i.e. on the morphological level, the clueword contains a negative prefix such as *in-* or *un-.* Here is a typical example:

In monocytic cells, IL-1beta treatment led to a production of ROIs which is independent of the 5-LOX enzyme but requires the NADPH oxidase activity.

The event denoted by *production* is not triggered by the presence of the 5-LOX enzyme, thus, there is no regulation event here and this is expressed in Genia by marking the regulation event with the attribute non-exist while in BioScope its meaning is considered to be lexicalized and not necessarily negative. Mismatches originating from morphological negation mostly include the adjective *independent.* We argue that although this word contains a negative prefix at the level of morphology, its meaning is lexicalized and not necessarily negative: it rather describes a state or a lack of relation between its arguments. In this way, it could be treated similarly to *lack*, that is, not the event itself but its state should be negated. On the other hand, cluewords including morphological negation can be easily identified by automatic methods (segmenting the word into a negative prefix and an existing (adjectival) morpheme) and these can be automatically tagged as negative cues.

The interpretation of some speculative keywords too seems to vary in BioScope and Genia Event. The most striking example is the case of events modified by other words or phrases expressing ability (e.g. *be able to*, *ability* etc.), which are annotated for probability in Genia but not in BioScope. An example is offered here:

NF-kappa B activation correlated with the ability of CD40 to induce Ab secretion and the up-regulation of ICAM-1 and LFA-1.

A highly interesting subclass of words expressing ability is when the derivational suffix conveys the ‘ability’ meaning as in *inducible* or *inhibitable.* Take the following sentence:

Despite stimulation with LPS, disruption of the NF-kappaB signaling pathway in precursor B cells led to the loss of inducible Oct-2 DNA binding activity in vitro and the suppression of Oct-2-directed transcription in vivo.

The event described by *inducible* can be paraphrased as *Oct-2 DNA binding activity can be induced in vitro*, which is an ‘ability’ usage of the auxiliary *can*, thus, it is annotated for probability in Genia but not in BioScope.

The lexical semantic-related differences originate from conceptual discrepancies of the two corpora. These mismatches can hardly be resolved without harmonizing the annotation principles behind the corpora and re-annotating the data. As one of the chief design goals of BioScope annotation was to be task-independent and the modality annotation of Genia is fine-tuned to biological event extraction, biological information extractors may incorporate the modality principles of Genia while BioScope annotations may be followed when the target domain differs from the biomedical one.

Lastly we note that few differences (about 5.7%) in annotation can be obviously traced back to annotation errors.

## Discussion

### Detailed event annotations

Table 1 and 2 reveal that the biggest subset of the differences (60%) came from the issue that Genia handles events within events as individual information sources while BioScope deals with constituent-based text spans. An interesting question for consideration is whether the expected output of an information extraction system consists of facts solely on the basis of this textual evidence, where the trigger for the event does not belong to the main statement of the sentence/document. Note that the information content of these events within events is usually introduced and discussed in detail in other parts of the document or in other publications or belongs to the trivial domain knowledge.

Similar considerations implied the design of the “Meta-Knowledge Annotation Scheme for Bio-Events” [21]. It introduces dedicated labeling dimensions of events about:

• New Knowledge (yes/no), the motivation of which is that events “...could correspond to new knowledge, but only if they represent observations from the current study, rather than observations cited from elsewhere. In a similar way, an analysis drawn from experimental results in the current study could be treated as new knowledge, but generally only if it represents a straightforward interpretation of results, rather than something more speculative.”

• Knowledge type (investigation / observation / analysis / general) whose “... purpose is to form the basis of distinguishing between the most critical types of rhetorical/pragmatic intent, according to the needs of biologists.”

Krallinger [22] also argues that from a biologist point of view only the events supported by experimental evidence are interesting. This entails that trivial domain knowledge and assertions without empirical evidence (i.e. weasels) should be treated distinctively. As the BioScope corpus is designed to be task-independent, its scopes could not be applied directly for the deep and detailed (sub)event annotation of Genia since many subevents that belong to trivial domain knowledge fall under scope. However, it can recognize the negation and hedge state of chief statements by exploiting syntactic relations (dependency links) between the keyword marked in BioScope and its trigger word (denoting the chief event): in this way it is possible to determine whether they represent new knowledge or not. Note that there are in-sentence scope detectors published and weasel detectors have been also created recently [11].

### BioScope for event modality detection

We discussed in the previous section that the scopes of BioScope are not useful directly to the detection of assertion and certainty state of Genia events, however, we believe that using cue phrases in event modality detection can yield significant contribution. For instance, Kilicoglu and Bergler [4] constructed lexicons for speculation and negation keywords and introduced rules for recognizing the modality state of an event by utilizing the dependency path between the event clue phrase and the speculation/negation cue. Kilicoglu and Bergler employed hand-crafted lexicons for cue recognition, however, keywords are ambiguous, i.e. they express speculation and negation just in certain contexts. Hence a cue phrase detection system is needed which classifies tokens based on their local context then the dependency paths between these predicted speculation/negation evidences and event triggers should be analyzed. The BioScope corpus can be employed as a training dataset for general speculation/negation cue classifiers. The state-of-the-art modifier cue detectors achieve strict phrase-level F-measures over 80% [11]. Dependency-based rules defined for each (sub)type of keywords can be also added to the system in order to determine the negative/speculative status of the event. As future work, we plan to develop an event modality detector which uses BioScope as a training database for identifying speculation/negation cues and is enhanced by hand-crafted dependency-based rules for determining the modality of the event.

### The usability of different annotation schemes

As discussed earlier, the annotation scheme of BioScope relies on linguistic principles while Genia Event is based on a more detailed annotation system specifically tailored to biological event annotation, where several complex relations are encoded between participants of the events – often across clause boundaries. In this way, the annotation scheme of Genia Event is highly domain-specific and the corpus can be fruitfully utilized in biomedical information extraction, resulting in a deep and precise analysis of biological events though it might require a lot of additional work to adapt the system to other domains. On the other hand, as the BioScope annotation scheme is linguistic-based, scope- and cue-marking rules extracted from the corpus data can be more easily exploited when developing negation/hedge detectors in other domains as well.

## Conclusions

In this paper, we discussed the differences between the linguistic-based and event-oriented annotation of negation and speculation in biological documents. We defined categories for the differences between the linguistic scope-based BioScope and the event-oriented Genia Event corpora. They have an intersection of documents (biological abstracts) which was randomly sampled, frequencies of mismatch categories were estimated and resolution strategies were also offered for them.

As far as information extraction in different domains is concerned, the annotation system in BioScope seems to be more easily adaptable to non-biomedical applications because of the high level of domain specificity in the Genia Event annotation system.

As regards the frequency of mismatch categories, we found that the largest amount of differences is due to the issue that scopes aim to identify the negation/certainty status of the key event in the sentence and each argument of these key events (including arguments that are events themselves) is under scope as well in BioScope. In contrast, Genia deals with the modality of events within events independently. The useful information for the biologist can be acquired from the key events, thus when detecting “new knowledge”, an automatic scope detector trained on BioScope can contribute to biomedical information extraction. On the other hand, BioScope cue phrases may be also employed to identify the assertion and certainty status of events. To reach this goal, we plan to develop a procedure which makes use of automatically recognized negation/speculation cues and employs syntax-based rules (investigating the dependency path between the modality cue and the event cue) to classify the status of the event.

## Competing interests

The authors declare that they have no competing interests.

## Authors contributions

VV and TO categorized the mismatches between the two corpora. GyM implemented the software tools for gathering and visualizing the differences. RF and GySz carried out the statistical analysis of mismatches.
